# Protist-Bacteria Associations: *Gammaproteobacteria* and *Alphaproteobacteria* Are Prevalent as Digestion-Resistant Bacteria in Ciliated Protozoa

**DOI:** 10.3389/fmicb.2016.00498

**Published:** 2016-04-11

**Authors:** Jun Gong, Yao Qing, Songbao Zou, Rao Fu, Lei Su, Xiaoli Zhang, Qianqian Zhang

**Affiliations:** ^1^Laboratory of Microbial Ecology and Matter Cycles, Yantai Institute of Coastal Zone Research, Chinese Academy of SciencesYantai, China; ^2^School of Life Science, South China Normal UniversityGuangzhou, China

**Keywords:** bacterial symbiosis, grazing-resistant bacteria, microbial interactions, protein secretion systems, top–down effect

## Abstract

Protistan bacterivory, a microbial process involving ingestion and digestion, is ecologically important in the microbial loop in aquatic and terrestrial ecosystems. While bacterial resistance to protistan ingestion has been relatively well understood, little is known about protistan digestion in which some ingested bacteria could not be digested in cells of major protistan grazers in the natural environment. Here we report the phylogenetic identities of digestion-resistant bacteria (DRB) that could survive starvation and form relatively stable associations with 11 marine and one freshwater ciliate species. Using clone library and sequencing of 16S rRNA genes, we found that the protistan predators could host a high diversity of DRB, most of which represented novel bacterial taxa that have not been cultivated. The localization inside host cells, quantity, and viability of these bacteria were checked using fluorescence *in situ* hybridization. The DRB were affiliated with *Actinobacteria, Bacteroidetes, Firmicutes, Parcubacteria* (OD1), *Planctomycetes*, and *Proteobacteria*, with *Gammaproteobacteria* and *Alphaproteobacteria* being the most frequently occurring classes. The dominance of *Gamma-* and *Alphaproteobacteria* corresponds well to a previous study of Global Ocean Sampling metagenomic data showing the widespread types of bacterial type VI and IV secretion systems (T6SS and T4SS) in these two taxa, suggesting a putatively significant role of secretion systems in promoting marine protist-bacteria associations. In the DRB assemblages, opportunistic bacteria such as *Alteromonadaceae, Pseudoalteromonadaceae*, and *Vibrionaceae* often presented with high proportions, indicating these bacteria could evade protistan grazing thus persist and accumulate in the community, which, however, contrasts with their well-known rarity in nature. This begs the question whether viral lysis is significant in killing these indigestible bacteria in microbial communities. Taken together, our study on the identity of DRB sheds new light on microbial interactions and generates further hypotheses including the potential importance of bacterial protein secretion systems in structuring bacterial community composition and functioning of “microbial black box” in aquatic environments.

## Introduction

Protistan grazing on bacteria is one of the most important ecological processes in microbial food webs that channel carbon and energy to higher trophic levels and regenerate nutrients (Azam et al., 1983). Typically, heterotrophic nanoflagellates (HNFs) are the primary grazers of bacteria, and ciliates can be significant bacterivores in eutrophic habitats (Sherr and Sherr, 2002). In the long evolutionary history of the interplay between bacterial preys and protistan predators, bacteria have seemingly developed many strategies to survive protistan grazing. These include: changes in cell size and filamentation, formation of aggregates, microcolonies and biofilms, increases of swimming speed, and chemical resistance to ingestion (for reviews see Jürgens and Güde, 1994; Hahn and Höfle, 2001; Jürgens and Matz, 2002; Matz and Kjelleberg, 2005; Pernthaler, 2005; Montagnes et al., 2008). It has been hypothesized the existence and development of predation-resistant bacteria may decrease of carbon and energy transfers in the microbial loop and limit nutrient regenerations (Jürgens and Güde, 1994).

Bacterial resistance to digestion represents another important means to survive protistan predation (Jürgens and Güde, 1994; Jousset, 2012). For example, certain *Synechococcus* and actinobacterial strains could not be digested by nanoflagellates (Boenigk et al., 2001; Zwirglmaier et al., 2009; Apple et al., 2011; Šimek et al., 2013). It was suggested that the presence of protective S-layer in the cell wall could protect *Synechococcus* cells from enzymatic degradation in food vacuoles of the ciliate *Tetrahymena* (Koval, 1993). Freshwater isolates of *Janthinobacterium lividum* and *Chromobacterium violaceum* could kill the nanoflagellate grazers by releasing a toxin (Matz et al., 2004). The pathogenic bacterium *Campylobacter jejuni* remained viable after ingestion for 5 h by a freshwater ciliate *Colpoda* sp. (First et al., 2012). Many bacterial strains (e.g., *Legionella, Listeria, Vibrio*, and *Salmonella*) could persist inside *Acantbamoeba* and *Tetrahymena* cells, which might have given rise to intracellular symbionts, parasites, and pathogens (Barker and Brown, 1994; Greub and Raoult, 2004; Brandl et al., 2005; Matz and Kjelleberg, 2005). However, these studies have mostly tested the digestibility of selected bacterial strains of pathogenicity and/or from freshwater environments. What has not been investigated much, so far, is the diversity and composition of the bacterial assemblages that are resistant to digestion by major protistan bacterivores in complicated microbial communities of aquatic systems.

Recently, we have investigated a range of ciliate species for identities of putatively DRB. Ciliates were chosen primarily because of their large cell size, which allowed to be easily manipulated at a single-cell level to minimize the chance of bacterial contaminations. Previously, we reported a new intracellular bacterial species belonging to the phylum *Parcubacteria* (the candidate division OD1) in a starved freshwater ciliate *Paramecium bursaria* (Gong et al., 2014). Here, we extend this line of research by identifying some DRB (and endosymbionts) in 11 marine and 1 freshwater ciliate species, with which we hope to provide a broad view of the diversity of bacterial populations that might have escaped from protistan digestion. The unveiled taxonomic affiliations of DRB enable us to link to enormous microbiological, genetic and ecological knowledge bearing on these bacterial names, which lays a basis to a better understanding of associations and interactions between bacteria and protists in marine microbial food webs, and to generate new ecological hypotheses.

## Materials and Methods

### Organisms, Source, and Culture Conditions

**FIGURE 1:**
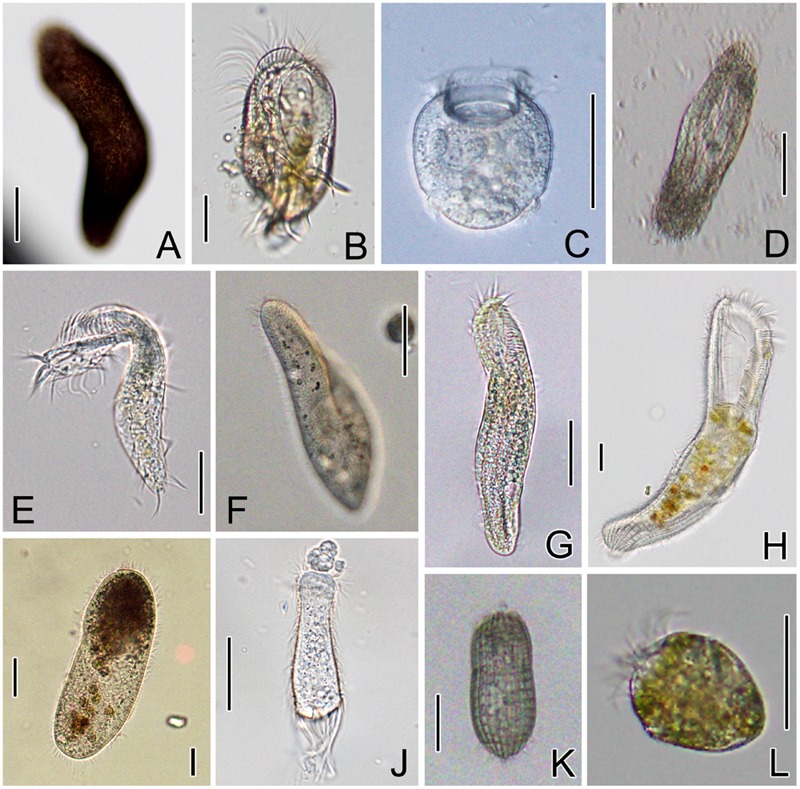
**Morphology of ciliate species *in vivo*. (A)** *Pseudokeronopsis carnea*; **(B)** *Diophrys scutum*; **(C)** *Urceolaria urechi*; **(D)** *Hemigastrostyla elongata*; **(E)** *Pseudokeronopsis flava*; **(F)** *Paramecium aurelia*; **(G)** *Uroleptopsis citrina*; **(H)** *Condylostoma spathiosum*; **(I)** *Cardiostomatella* sp.; **(J)** *Boveria labialis*; **(K)** *Coleps* sp.; **(L)** *Strombidium sulcatum.* Scale bars = 50 μm.

Thirteen strains of 12 (11 marine and 1 freshwater) ciliate species belonging to four classes, Spirotrichea, Oligohymenophorea, Heterotrichea, and Prostomatea, were investigated (**Table 1**). Ten free-living species were kindly provided by Prof. Weibo Song’s lab at Ocean University of China (OUC), Qingdao, or directly sampled from aquatic environments by the authors of this work. Two endosymbiotic ciliates, *Boveria labialis* and *Urceolaria urechi*, were isolated from the sea cucumber (*Apostichopus japonicas*) and the Chinese penis fish (*Urechis unicinctus*), respectively. The host animals were purchased from local seafood markets in Yantai. Seven cultured strains were maintained in Petri dishes at 18°C for several days, with water from the sampling sites and several rice grains to enrich bacteria for food (**Table 1**). All ciliates were observed *in vivo* for living features (**Figure 1**), and identified according to the taxonomic reference (Song et al., 2009).

**Table 1:** A summary of ciliate species investigated in this study.

Ciliate	Classification	Habitat/host	Original location	Provider	Culture
*Boveria labialis*	Oligohymenophorea	Sea cucumber (*Apostichopus japonicas*)	Culturing ponds, Yantai	Market	No
*Cardiostomatella* sp.	Oligohymenophorea	Marine	Littoral sediment, Yantai	The authors	No
*Coleps* sp.	Prostomatea	Marine	Estuarine water, Yantai	The authors	No
*Condylostoma spathiosum* WL	Heterotrichea	Marine	Sandy beach, Yantai	The authors	No
*Condylostoma spathiosum* ZS	Heterotrichea	Marine	Waste water discharge, Yantai	The authors	No
*Diophrys scutum*	Spirotrichea	Marine	Littoral zone, Yantai	The authors	Yes
*Hemigastrostyla elongata*	Spirotrichea	Marine	Littoral zone, Yantai	The authors	Yes
*Paramecium aurelia*	Oligohymenophorea	Freshwater	A small pond, Qingdao	OUC	Yes
*Pseudokeronopsis carnea*	Spirotrichea	Marine	Littoral zone, Qingdao	OUC	Yes
*Pseudokeronopsis flava*	Spirotrichea	Marine	Littoral zone, Qingdao	OUC	Yes
*Strombidium sulcatum*	Spirotrichea	Marine	Littoral zone, Yantai	The authors	Yes
*Urceolaria urechi*	Oligohymenophorea	Chinese penis fish (*Urechis unicinctus*)	Littoral sediment, Yantai	Market	No
*Uroleptopsis citrina*	Spirotrichea	Marine	Littoral zone, Qingdao	OUC	Yes

### DNA Extraction, Clone Libraries, and Sequencing

Ciliate cells were transferred into autoclaved seawater (sterilized double distil water for the freshwater species *Paramecium aurelia*) with a micropipette. In order to minimize contaminations, cells were washed for three to five times to remove microorganisms attaching cilia and cell surface. The ciliates were then maintained in the sterilized water for 12 to 24 h, allowing the starving hosts to digest the ingested bacteria as much as possible. After starvation, the remaining individuals were washed again. Three to five individuals were transferred to a PCR tube with a minimum volume of water for DNA extraction, and up to 20 individuals were mounted onto slides for subsequent fluorescence *in situ* hybridization (FISH) assays.

Genomic DNA extraction was performed as previously described (Gong et al., 2014). Bacterial 16S rRNA genes were PCR amplified with primer set 8F (5′- AGAGTTTGATCCTGGC TCAG -3′) and 1492R (5′-GGTTACCTTGTTACGACTT-3′), or with 8F and 1392R (5′- ACGGGCGGTGTGTAC -3′) (Lane, 1991). The PCR reaction solution (25 μl) contained 1 μl of 10 μM primers, 1 μl extracted DNA solution, 2.5 μl dNTP mix (0.2 mM of each) and 0.625 units of DreamTaq DNA polymerase and 2.5 μl 10X DreamTaq buffer with MgCl_2_ at a concentration of 20 mM (Thermo Scientific, USA). All PCR reactions were performed in a Biometra thermal cycler with the following program: an initial denaturation 94°C for 3 min, followed by 34 cycles of 94°C for 1 min, annealing (at 50°C for primers 8F/1492R, and 52°C for primers 8F/1392R) for 1 min, and 72°C for 1 min, with a final extension step of 72°C for 10 min. The amplified PCR products were purified with a gel purification kit (Tiangen Biotech, China), ligated into pTZ57R/T vector using InsTAclone PCR Clone Kit (Thermo Scientific) and transformed into competent cells of *Escherichia coli* DH5α. The clones containing the DNA inserts were randomly selected. These positive clones were either pre-screened using restricted fragment length polymorphism (RFLP) analysis with two (*Taq* and *Hha*I, or *Taq* and *Msp*I) or one restricted enzyme (*Msp*I) (FastDigest, Thermo Scientific, USA), or directly sent for sequencing on an ABI 377 automated sequencer (Sangon, Shanghai, China). A total of 13 clone libraries of bacterial 16S rRNA genes were constructed for the 13 ciliate strains.

### Phylogenetic Analysis

The newly obtained 16S rRNA gene sequences were first aligned using MAFFT v.7 (Katoh and Standley, 2013). Chimeric sequences were identified using Bellerophon (Huber et al., 2004), and then removed for the subsequent analyses. The remaining sequences were subjected to BLAST against GenBank, and to ribosomal database project (RDP) databases for classification (Cole et al., 2009). Closely related sequences were retrieved from GenBank and aligned with these newly obtained. The compiled sequences were then aligned using SINA (ARB-Silva) with default settings (Pruesse et al., 2012) and manually modified, resulting in a final alignment of 1,365 positions. Maximum likelihood (ML) trees were constructed with FastTree V.2 program by default settings (Price et al., 2010), under a GTR+CAT model. The resulted ML tree were further organized and revised by Interactive Tree of Life (iTOL)^1^.

For some sequences that assigned into unclassified *Gammaproteobacteria, Rickettsiales*, and *Flavobacteria* by the RDP classifier, both ML and Bayesian inference (BI) analyses were carried out to further resolve their taxonomic ranks. PhyML program was used for building a ML tree under a best-fit GTR+G+I model. BI analyses were performed with MrBayes 3.1.2 (Ronquist and Huelsenbeck, 2003). Markov chain Monte Carlo (MCMC) simulations were run with two sets of four chains using the default settings, with a sampling frequency of 0.01. Convergence of the chain length was confirmed from the standard deviation of split frequencies (<0.01). 1,000,000 or 2,000,000 generations were run for these datasets. Twenty-five percent of generations were discarded as burn-in in each analysis. To characterize the “species”-level composition and variations of the DRB assemblages, operational taxonomic units (OTUs) were defined at a cutoff of 97% sequence similarity and analyzed using the Mothur program (Schloss et al., 2009).

To explore the beta diversity of DRB among ciliate specimens, a Bray–Curtis similarity matrix was calculated based on the relative abundance of different families, and visualized with the Clustering method using the software PRIMER 6 (PRIMER-E, UK). Differences in assemblage structure among samples were statistically tested using analysis of similarity (ANOSIM) (Clarke and Gorley, 2006), to examine the possible effect of habitat (marine vs. freshwater), class-level taxonomy (four classes), life style (free-living vs. symbiotic), and sampling method (environmental isolate vs. laboratory culture) of the hosts.

### Probe Design and Fluorescence *In Situ* Hybridization

A 16S rRNA-targeted oligonucleotide probe targeting the genus *Aestuariibacter*, which included the most common DRB phylotypes in this study, was designed as previously described (Gong et al., 2014). In brief, conserved 16S rRNA regions of *Aestuariibacter* species were identified based on the rRNA alignment of a range of species. Several short fragments (length of 16–20 nucleotides) in these regions were then selected and evaluated using PROBE MATCH of RDP release 10 (Cole et al., 2009). A web tool, mathFISH, was used for assessing sensitivity and specificity, and the optimum formamide concentration (40%) for mismatch discrimination optimizing (Yilmaz et al., 2011). The newly designed probe was named ALT658, with the sequence 5′-TTCCACTCCCCTCTCCAA-3′.

A number of ciliates examined in this study were subjected to FISH with a mixture of universal eubacterial probes, EUB338, II and III (Amann et al., 1995; Daims et al., 1999). The non-sense probe NON338, complementary to EUB338, was used as a negative control for the hybridization protocol (Manz et al., 1992). In the case of detection of *Aestuariibacter* phylotypes in ciliate hosts, FISH with the probe ALT658 was performed separately to reveal the quantity and location of the bacteria. All probes in this study were labeled with Cy3 at the 5′ end.

Whole-cell hybridization was according to (Fried et al., 2002). Cells were fixed with Bouin’s solution (50%, final concentration). Cell suspensions were dropped onto microscopic slides (SuperFrost Plus) and air dried at room temperature. The slides were put away in a black box and stored at 4°C. Before FISH assay, the slides were washed in distilled water three times for 10 min and then progressively dehydrated via an ethanol gradient (30, 50, 80, and 100%). Slides were incubated at 46°C for 3 h in hybridization buffer, which contained 20 mM Tris-HCl (pH 8.0), 0.9 M NaCl, 0.01% sodium dodecyl sulphate (SDS), 30% (40% for the probe ALT658) formamide and the relevent fluorescent probe (5 ng μl^-1^ final concentration). After hybridization, slides were washed for 15 min at 48°C with wash buffer [20 mM Tris-HCl (pH 8.0), 450 mM NaCl, 0.01% SDS], and then rinsed with chilled Milli-Q water. Slides mounted with anti-fade mounting medium (Beyotime, China) and DAPI (50 ng ml^-1^) were observed under an epifluorescence microscope (Olympus BX51, Japan) with green-light excitation (wavelength 505 to 560 nm) for Cy3 and UV excitation (wavelength 330 to 385 nm) for DAPI signals. A SPOT RT3 digital camera (SPOT Imaging Solutions, Sterling Heights, USA) was used for visualization.

### Sequence Accession Numbers

The bacterial 16S rRNA gene sequences newly obtained in this study have been deposited in the GenBank database under the accession numbers KU524761 – KU524878.

## Results

### Diversity and Composition of Digestion-Resistant Bacteria (DRB)

**FIGURE 2:**
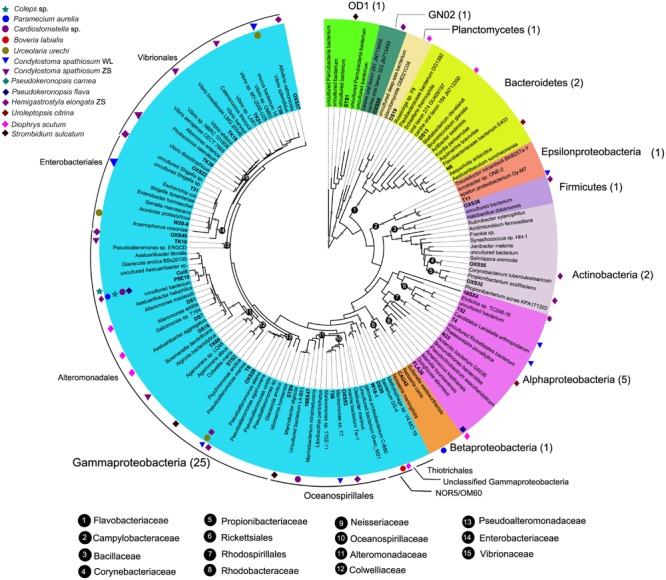
**A maximum likelihood (ML) tree of 16S rRNA genes showing the phylogeny of digestion-resistant bacteria (DRB) detected in 12 species of ciliated protists.** Representative sequences of 40 bacterial OTUs were used to build the tree under a GTR+CAT model. Numbers in parentheses indicate the number of bacterial OTUs affiliated with the phyla/candidate divisions. Note that OTUs of *Gammaproteobacteria* (*Alteromonadaceae, Pseudoalteromonadaceae*, and *Vibrionaceae*) and *Alphaproteobacteria* represent the most abundant digestion-resistant bacterial groups.

A total of 118 bacterial 16S rRNA gene sequences were obtained from clone libraries constructed for DRB in 12 ciliate species. Classification via RDP classifier pipeline showed that the digestion-resistant bacteria (DRB) were highly diverse. There were 40 OTUs, which were affiliated with 13 identified families of seven bacterial phyla/candidate divisions, *Actinobacteria, Bacteroidetes, Firmicutes*, GN02, *Planctomycetes, Proteobacteria* (including four classes: *Alpha*-, *Beta*-, *Gamma*-, and *Epsilonproteobacteria*), and *Parcubacteria* (OD1) (**Figure 2**; **Table 2**).

**Table 2:** The closest matches in the Genbank by BLASTing 16S rRNA genes and ribosomal database project (RDP) classification of bacteria associated with ciliate host species subjected to starvation.

OTU ID	Closest matched species (accession number)	Coverage (%)	Identity (%)	Classification	Ciliate host
OTU1	*Aestuariibacter halophilus* strain JC2043 (AY207503)	97-98	94-95	*Gamma, Alteromonadaceae*	*Cardiostomatella* sp., *Coleps* sp., *Paramecium aurelia, Pseudokeronopsis carnea, Pseudokeronopsis flava*
OTU2	*Aestuariibacter halophilus* strain JC2043 (AY207503)	97	94	*Gamma, Alteromonadaceae*	*Coleps* sp.
OTU3	*Alteromonas* sp. KU27F1 (AB636144)	99	98	*Gamma, Alteromonadaceae*	*Diophrys scutum*
OTU4	*Alteromonas* sp. H86 (FJ903192)	99	98-99	*Gamma, Alteromonadaceae*	*Diophrys scutum*
OTU5	*Alteromonas* sp. CF11-5 (FJ170012)	100	99	*Gamma, Alteromonadaceae*	*Diophrys scutum*
OTU6	*Agarivorans* sp. LQ48 (FJ593496)	99	99	*Gamma, Alteromonadaceae*	*Condylostoma* sp. ZS
OTU7	*Marinobacter algicola* strain DG893 (NR_042807)	92	99	*Gamma, Alteromonadaceae*	*Strombidium sulcatum*
OTU8	*Pseudoalteromonas* sp. BSw20582 (EF639360)	100	99-100	*Gamma, Pseudoalteromonadaceae*	*Condylostoma spathiosum* WL, *Hemigastrostyla elongata, Urceolaria urechi*
OTU9	*Pseudoalteromonas* sp. BSw21650 (JF697294)	100	97	*Gamma, Pseudoalteromonadaceae*	*Hemigastrostyla elongata*
OTU10	*Pseudoalteromonas* sp. ERGCD (HM439609)	99	96	*Gamma, Pseudoalteromonadaceae*	*Condylostoma spathiosum* ZS
OTU11	*Vibrio harveyi* strain ATCC 33843 (392 [MAV]) (CP009467)	99	95	*Gamma, Vibrionaceae*	*Condylostoma spathiosum* ZS
OTU12	*Vibrio* sp. MY-2008-U28 (FM957470)	99	99	*Gamma, Vibrionaceae*	*Condylostoma spathiosum* ZS
OTU13	*Vibrio brasiliensis* strain LMG 20546 (NR_025477)	98	99	*Gamma, Vibrionaceae*	*Condylostoma spathiosum* ZS
OTU14	*Vibrio splendidus* LGP32 (FM954972)	99	99	*Gamma, Vibrionaceae*	*Condylostoma spathiosum* WL, *Urceolaria urechi*
OTU15	*Vibrio diazotrophicus* strain N6 (JF775501)	100	99	*Gamma, Vibrionaceae*	*Hemigastrostyla elongata*
OTU16	*Vibrio diazotrophicus* strain N6 (JF775501)	100	95	*Gamma, Vibrionaceae*	*Hemigastrostyla elongata*
OTU17	*Escherichia coli* W (CP002185)	100	99	*Gamma, Enterobacteriaceae*	*Condylostoma spathiosum* WL
OTU18	*Raoultella ornithinolytica* B6 (NR_102983)	100	99	*Gamma, Enterobacteriaceae*	*Urceolaria urechi*
OTU19	*Enterobacter hormaechei* skg0061 (HQ322393)	100	94	*Gamma, Enterobacteriales*	*Hemigastrostyla elongata*
OTU20	*Oceanospirillum beijerinckii* NBRC 15445 (NR_113754)	97	99	*Gamma, Oceanospirillaceae*	*Cardiostomatella* sp.
OTU21	*Marinomonas* sp. 170Z-11 (JX310217)	99	98	*Gamma, Oceanospirillaceae*	*Condylostoma spathiosum* WL
OTU22	*Marinomonas* sp. S3726 (FJ457289)	99	97	*Gamma, Oceanospirillaceae*	*Hemigastrostyla elongata*
OTU23	*Colwellia psychrerythraea* ATCC 27364 (NR_037047)	91	98	*Gamma, Colwelliaceae*	*Strombidium sulcatum*
OTU24	*Haliea* sp. ETY-NAG (AB646260)	99	93	*Gamma*, NOR5/OM60	*Boveria labialis*
OTU25	*Spongiibacter marinus* strain DSM 17750 (NR_118015)	99	99	Gamma, unclassified	*Diophrys scutum*
OTU26	*Holospora undulata* (HE797906)	82	81-82	*Alpha, Rickettsiales*	*Cardiostomatella* sp.
OTU27	*Candidatus* Lariskella arthropodarum (JQ726733)	97	89	*Alpha, Rickettsiales*	*Condylostoma spathiosum* WL
OTU28	Endosymbiont of *Acanthamoeba* sp. UWC8 (CP004403)	99	88	*Alpha, Rickettsiales*	*Condylostoma spathiosum* WL
OTU29	*Donghicola eburneus* strain SW-277 (NR_043928)	98	99	*Alpha, Rhodobacteraceae*	*Diophrys scutum, Pseudokeronopsis flava*
OTU30	*Thalassospira povalilytica* strain Zumi 95 (NR_125450)	97	99	*Alpha, Rhodospirillaceae*	*Uroleptopsis citrina*
OTU31	*Neisseria flavescens* strain OH1033A (KF030235)	99	99	*Beta, Neisseriaceae*	*Paramecium aurelia*
OTU32	*Arcobacter* sp. ONE-2 (KF650753)	100	98	*Epsilon, Campylobacteraceae*	*Condylostoma spathiosum* WL
OTU33	*Propionibacterium acnes* KPA171202 (NR_074675)	99	99	*Actinobacteria, Propionibacteriaceae*	*Hemigastrostyla elongata*
OTU34	*Corynebacterium* sp. CIP107067 (AJ438051)	99	99	*Actinobacteria, Corynebacteriaceae*	*Hemigastrostyla elongata*
OTU35	*Chryseobacterium hominis* NF802 (NR_042517)	99	99	*Bacteroidetes, Flavobacteriaceae*	*Diophrys scutum*
OTU36	*Frondibacter aureus* (AB932598)	95	95	*Bacteroidetes, Flavobacteriaceae*	*Uroleptopsis citrina*
OTU37	*Halobacillus* sp. FAM 114 (JN381955)	99	87	*Firmicutes, Bacillaceae*	*Hemigastrostyla elongata*
OTU38	Planctomycete GMD21C08 (AY162119)	93	95	*Planctomycetes, Phycisphaeraceae*	*Diophrys scutum*
OTU39	Bacterium SH4-10 (JQ269257)	91	81	OD1 (Parcubacteria)	*Strombidium sulcatum*
OTU40	GN02 bacterium 323 clone 1D068 (JN713493)	95	88	GN02	*Hemigastrostyla elongata*

*Alpha, Beta, Gamma, and Epsilon indicate the bacterial classes Alphaproteobacteria, Betaproteobacteria, Gammaproteobacteria, and Epsilonproteobacteria, respectively.*

#### Gammaproteobacteria

Overall, *Gammaproteobacteria* phylotypes dominated the DRB assemblages. About 63% (25) observed OTUs of the DRB were affiliated with this class (**Figure 2**; **Table 2**). These included *Alteromonadaceae* (7 OTUs), *Pseudoalteromonadaceae* (3 OTUs), *Vibrionaceae* (6 OTUs), *Enterobacteriaceae* (3 OTUs), *Oceanospirillaceae* (3 OTUs), *Colwelliaceae* (1 OTU), the NOR5/OM60 group (1 OTU) and an unclassified OTU within the class.

**FIGURE 3:**
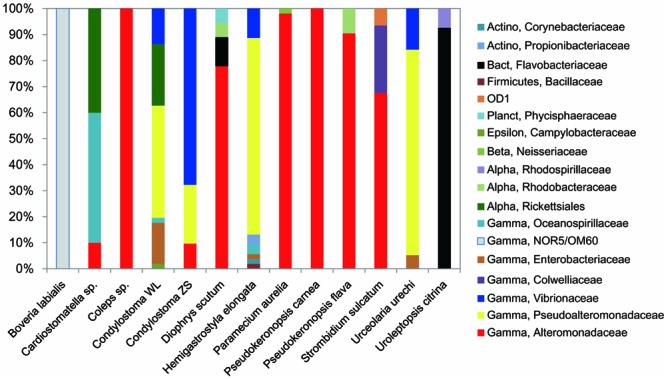
**Relative abundances of major taxa in digestion-resistant bacterial assemblages in 12 species (13 strains) of ciliates.** *Actinobacteria* (Actino), *Alphaproteobacteria* (Alpha), *Bacteroidetes* (Bact), *Betaproteobacteria* (Beta), *Gammaproteobacteria* (Gamma), *Epsilonproteobacteria* (Epsilon), *Firmicutes, Parcubacteria* (OD1), and *Planctomycetes* (Planct) were detected. Taxa are classified at the family level unless it is not supported (<a threshold of 80%) by ribosomal database project (RDP) classifier pipeline. Note that the families *Alteromonadaceae, Pseudoalteromonadaceae*, and *Vibrionaceae* are the most frequently occurred and generally account for a large proportion in the assemblages.

*Alteromonadaceae* phylotypes (OTU1–OTU7) were observed in eight ciliate strains, being the most frequently occurred family in the DRB assemblages (**Figures 2** and **3**; **Table 2**). A single OTU of this family (OTU1), which exhibited 94–95% similarities to *Aestuariibacter halophilus* strain JC2043 in 16S rRNA gene sequences, was detected coincidently in five ciliate species, with higher proportions in *Coleps* sp. (100%), *Pseudokeronopsis carnea* (100%), *Paramecium aurelia* (96%), and *Pseudokeronopsis flava* (90%) than in *Cardiostomatella* sp. (10%). Other phylotypes of this family were closely related to *Alteromonas* sp. (98–99% similarities) and *Marinobacter algicola* (99%), and found with high proportions in the DRB assemblages of *Diophrys scutum* (78%) and *Strombidium sulcatum* (52%), respectively. The *Agarivorans* phylotype occurred only in *Condylostoma spathiosum* strain ZS with a proportion of 10% (**Figures 2** and **3**; **Table 2**).

*Pseudoalteromonadaceae* phylotypes (OTU8–OTU10) in the DRB assemblages showed 95–100% sequence similarities with members of *Pseudoalteromonas*. These phylotypes occurred with high proportions in the DRB assemblages of *Urceolaria urechi* (79%) and *Hemigastrostyla elongata* (68%), but lower in the two strains of *Condylostoma spathiosum* (43 and 23%) (**Figures 2** and **3**; **Table 2**).

*Vibrionaceae* phylotypes (OTU11–OTU16) were frequently detected among the DRB assemblages of three ciliate species (four strains) (**Figure 3**). These phylotypes shared 95–99% sequence similarities with vibrios (**Table 2**). The highest proportion (68%) of *Vibrionaceae* was recorded in *Condylostoma spathiosum* strain ZS. However, much lower proportions of vibrios were observed in the *Condylostoma* strain WL (14%), *Hemigastrostyla elongata* (10%), and *Urceolaria urechi* (16%) (**Table 2**).

*Enterobacteriaceae* phylotypes (OTU17–OTU19) were detected with relatively lower proportions in the DRB assemblages of *Condylostoma spathiosum* strain WL (16%), *Urceolaria urechi* (5%), and *Hemigastrostyla elongata* (2%) (**Figure 3**). BLAST against GenBank indicated that these phylotypes were closely related to *Escherichia coli, Enterobacter hormaechei*, or *Raoultella ornithinolytica*, with sequence similarities of 94 and 99%. *Hemigastrostyla elongata* hosted the OTU19, which was affiliated with the order *Enterobacteriales* (**Table 2**).

Three OTUs of *Oceanospirillaceae* (OTU20–OTU22) likely representing species of *Oceanospirillum* and *Marinomonas* were detected in three ciliate species with variable proportions: *Cardiostomatella* sp. (50%), *Condylostoma spathiosum* WL (2%), and *Hemigastrostyla elongata* (3%) (**Figure 3**; **Table 2**). A *Colwelliaceae* OTU closely related to *Colwellia psychrerythraea* (98% sequence similarity) accounted for 20% in the DRB assemblage of *Strombidium sulcatum* (**Figure 3**; **Table 2**).

**FIGURE 4:**
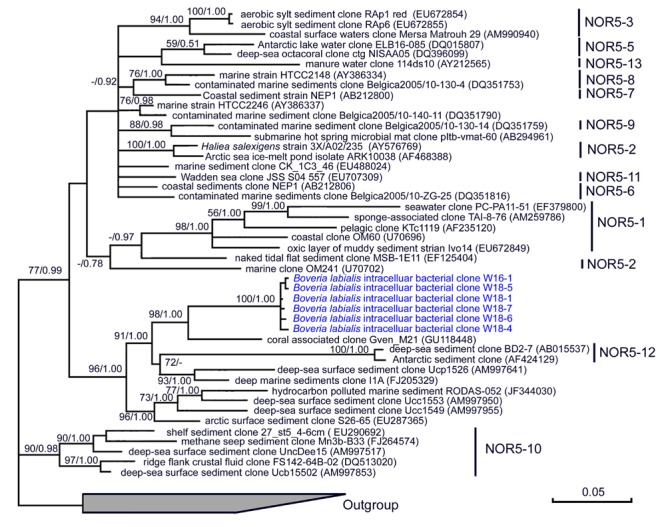
**A consensus tree based on 16S rRNA genes showing the phylogenetic position (class *Gammaproteobacteria*, group NOR5/OM60) of a bacterium detected in the macronucleus and micronucleus of ciliate *Boveria labialis*, an endosymbiotic protist from sea cucumbers.** The trees were constructed using ML (under GTR+G+I model) and Bayesian algorithms. Bootstrap values lower than 50% and posterior probability lower than 0.5 were not shown.

Ribosomal database project pipeline assigned two OTUs (OTU24 and OTU25) into the class *Gammaproteobacteria*, but lower rank classification could not be resolved. The OTU24 were only observed in *Boveria labialis*, showing a sequence identity of 93% to *Haliea* sp. ETY-NAG, an ethylene-assimilating marine bacterium (Suzuki et al., 2012). Nevertheless, our phylogenetic analyses further demonstrated that OTU24 was affiliated to an uncultured gammaproteobacterial group called NOR5/OM60, and closely related to the members of subclade NOR5–12 (see **Figure 4**), of which sequences are mainly from deep-sea samples and cultured members are known as aerobic anoxygenic phototrophs that need organic substrates like carboxylic acids, oligopeptides, or fatty acids for growth (Fuchs et al., 2007; Yan et al., 2009). OTU25 detected in *Diophrys scutum* ciliates could represent a strain of *Spongiibacter marinus* (99% similarity) (**Table 2**), a gammaproteobacterium originally isolated from a marine sponge (Graeber et al., 2008).

#### Alphaproteobacteria

Five out of the 40 OTUs (13%) of DRB found in this study were affiliated to the class *Alphaproteobacteria*. The *Donghicola eburneus*-like OTU29 (family *Rhodobacteraceae*) occurred in the two ciliates *Pseudokeronopsis flava* and *Diophrys scutum* with minor proportions (<10%) (**Figure 3**; **Table 2**). The OTU (OTU46) of the family *Rhodospirillaceae* was *Thalassospira povalilytica*-like (99% similarity), occurred only in the ciliate *Uroleptopsis citrina* (**Table 2**).

**FIGURE 5:**
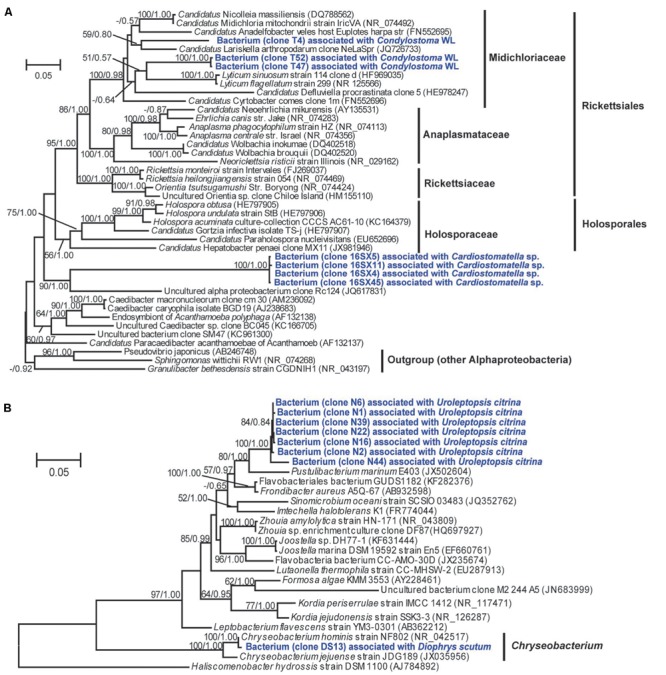
**Consensus trees based on 16S rRNA genes showing the phylogenetic positions of digestion-resistant bacterial phylotypes affiliated with the order *Rickettsiales***(A)** and the family *Flavobacteriaceae***(B)**.** The trees were constructed using ML (under GTR+G+I model) and Bayesian algorithms. Bootstrap values lower than 50% and posterior probability lower than 0.5 were not shown.

Three alphaproteobacterial OTUs (OTU26–OTU28) were assigned with the order *Rickettsiales* by RDP classifier (**Table 2**). Phylogenetic analyses showed that the two OTUs (OTU27 and OTU28) detected in *Condylostoma spathiosum* WL were closely related to a symbiont of *Acanthamoeba* sp. (88% similarity) and *Candidatus* Lariskella arthropodarum (89% similarity), and placed well within *Midichloriaceae* (ML 100%, BI 0.98) (see **Figure 5A**), one of three families of the order *Rickettsiales* (Ferla et al., 2013). The remaining OTU (OTU26) sharing 81–82% sequence similarities with *Holospora undulata* represented one of the basal lineages to *Rickettsiales* and *Holosporales* (**Figure 5A**). OTU26 occurred only in the ciliate *Cardiostomatella* sp. with a moderate proportion (40%).

#### *Betaproteobacteria* and *Epsilonproteobacteria*

Phylotypes of these two classes were rarely detected in the DRB assemblages. The freshwater ciliate *Paramecium aurelia* hosted a bacterial phylotype (OTU31) of *Neisseria flavescens* (99% similarity), a member of the family *Neisseriaceae*. OTU32, an epsilonproteobacterial phylotype detected once *Condylostoma spathiosum* WL, appeared to be a member of the genus *Arcobacter* (98%), family *Campylobacteraceae* (**Table 2**).

#### Actinobacteria

Two actinobacterial OTUs (OTU33 and OTU34) were classified into two families, *Corynebacteriaceae* and *Propionibacteriaceae*, which all were recovered from the hypotrich ciliate *Hemigastrostyla elongata* (**Table 2**). These sequences resemble most to these of *Propionibacterium acnes* and *Corynebacterium acnes* (99%) and *Propionibacterium* sp. (99%), respectively. Nevertheless, the proportion of *Actinobacteria* in the DRB assemblages was much lower (3%) (**Figure 3**).

#### Bacteroidetes

There were two bacteroidetes OTUs (OTU35, OTU36), all of which were affiliated to the family *Flavobacteriaceae*. OTU35 was associated with *Diophrys scutum*, representing the species *Chryseobacterium hominis* (99% similarity) (**Figure 5B**; **Table 2**). OTU36 was closely related to *Frondibacter aureus* (95% similarity) and appeared with a high proportion (93%) in the ciliate *Uroleptopsis citrina* (**Table 2**).

#### *Firmicutes, Planctomycetes, Parcubacteria* (OD1), and GN02

Digestion-resistant bacteria affiliated with these phyla/candidate divisions were generally minor in the assemblages. *Hemigastrostyla elongata* hosted phylotypes of *Bacillaceae (Firmicutes)* (OTU37) and the candidate division GN02 (OTU40), which shared low sequence identities with *Halobacillus* sp. (87%) and a GN02 bacterium (88%), respectively (**Table 2**; **Figure 3**). Also, a phylotype (OTU38) of *Phycisphaeraceae* (*Planctomycetes)* was found in *Diophrys scutum*, showing a sequence identity of 95% with a cultured planctomycete (**Table 2**). One of bacterial phylotypes from the ciliate *Strombidium sulcatum* belonged to *Parcubacteria* (OD1), of which many sequences await to be further designated to lower taxonomic ranks. This DRB phylotype showed a closest match (90% identity) with an environmental sequence from a trichloroethylene-contaminated aquifer (**Table 2**), but had no signature sequence fragment of OD1-p, a clade previously recognized within the candidate division (Gong et al., 2014).

### Beta Diversity of DRB

**FIGURE 6:**
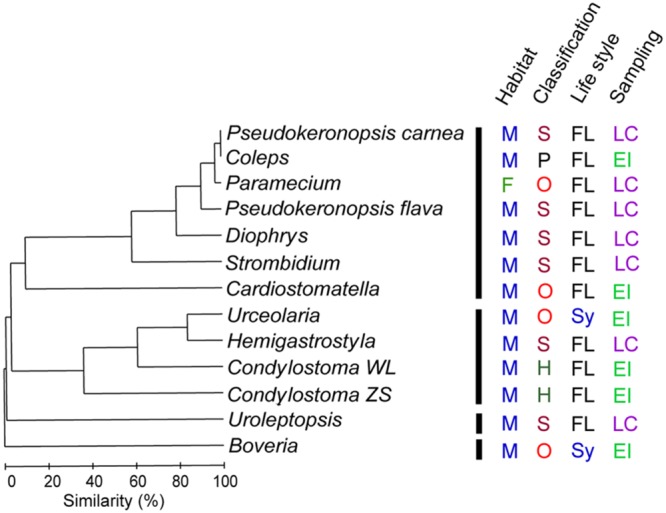
**Clustering of digestion-resistant bacterial (DRB) assemblages among 13 ciliate specimens.** Based on the distribution of each bacterial families detected, the assemblages can be divided into four groups at a similarity level of 10%. This grouping pattern of DRB composition hardly reflects differences between marine (M) and freshwater (F) habitats, class-level affiliations (Heterotrichea, H; Oligohymenophorea, O; Prostomatea, P; and Spirotrichia, S), and sampling sources (environmental isolation, EI and laboratory culture, LC) of the hosts. Nevertheless, two symbiotic (Sy) ciliates appeared to have significantly different DRB compared with those free-living (FL) species (ANOSIM, *P* < 0.05).

Since few OTUs were in common among DRB assemblages of ciliate species, we calculated their beta diversity based on the relative abundance variations of phylotypes at the family level. At the level of 10% similarity, the 13 DRB assemblages were clustered into four groups (**Figure 6**). The DRB turnover appeared to be high and did not follow a specific pattern with respective to the taxonomic affiliation (ANOSIM, *R* = -0.045, *P* = 0.578), habitat (*R* = -0.24, *P* = 0.923), or the sampling source (*R* = 0.106, *P* = 0.133). However, compared with the free-living species, the two symbiotic ciliates (*Boveria labialis* and *Urceolaria urechi*) hosted significantly different DRB assemblages (*R* = 0.44, *P* = 0.026).

### Fluorescence *In Situ* Hybridization

Searching through Probe Match function of RDP showed that the newly designed probe ALT658 completely matched 4273 sequences in the database, among which 4223 sequences (98.8%) were affiliated with the family *Alteromonadaceae* (13694 sequences curated in RDP), particularly the three genera of the family: *Aestuariibacter* (407/524), *Alteromonas* (2715/4114), and *Glaciecola* (621/759) (accessed on January 3, 2016). This indicates that the probe ALT658 is of high specificity and targets all *Alteromonadaceae* bacterial taxa recovered in this study, though not all known members of *Alteromonadaceae* could be matched.

**FIGURE 7:**
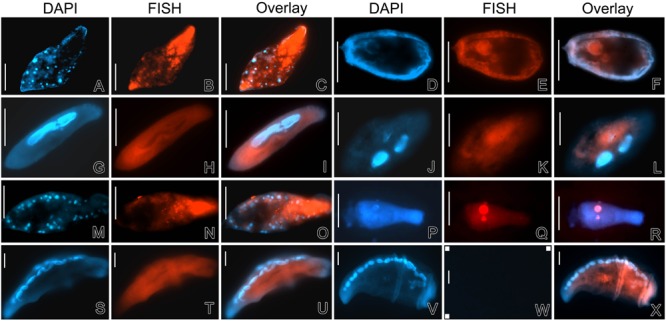
**Detection of DRB in ciliates using fluorescence in situ hybridization (FISH) and DAPI staining. (A–C)** *Pseudokeronopsis flava*; **(D–F)** *Coleps* sp.; **(G–I)** *Paramecium aurelia*; **(J–L)** *Hemigastrostyla elongata*; **(M–O)** *Uroleptopsis citrina*; **(P–R)** *Boveria labialis*; **(S–U)** *Condylostoma spathiosum* strain WL; **(V–X)** *Condylostoma spathiosum* strain ZS. The specimens were DAPI-stained and excited with UV, focusing on the nuclear apparatus **(A,D,G,J,M,P,S,** and **V)**. FISH was performed with Cy3-labeled probe ALT658 targeting members of the family *Alteromonadaceae* in ciliates *Coleps* sp., *Uroleptopsis citrina*, and *Paramecium aurelia*; other specimens were examined with universal eubacterial probes (EUB338, II and III). **(W)** Shows an example of negative controls by using the non-sense probe Cy3-NON338 in the FISH protocol, in which neither Cy3 fluorescence nor autofluorescence could be observed. Scale bars = 50 μm.

Fluorescence *in situ* hybridization with eubacterial probes (EUB338, II, and III) and the *Alteromonadaceae*-specific probe ALT658 revealed that most of the targeted bacteria presented in the cytoplasm of ciliate cells (**Figure 7**). In *Uroleptopsis citrina* and *Pseudokeronopsis flava* ciliates, red fluorescence of Cy3-labeled probes was relatively stronger, mostly presented as aggregates and positioned at the anterior portion of the host cells, indicating the DRB were active (**Figures 7A–C,M–O**). However, in other ciliate species such as *Paramecium aurelia* and two strains of *Condylostoma spathiosum*, the positive signal was relatively weak, diffuse and/or irregularly distributed in the ciliate cytoplasm (**Figures 7G–L,S–X**). In all cases, the negative controls with the non-sense probe NON338 were applied, and no Cy3-labeled signal was observed (see **Figure 7W**); no obvious food vacuoles were observed in starved and fixed specimens either.

There were positive FISH signatures inside the cell and on the surface of the ciliate *Coleps* sp. (**Figures 7D–F**), indicating some of the associated bacterial individuals were indeed intracellular, but others might be contamination or of ectosymbiontic nature. Nevertheless, all these bacteria (OTU1 and OTU2) associating with the *Coleps* species appeared to be closely related to *Aestuariibacter.* In the endosymbiotic ciliate *Boveria labialis*, the bacteria (belonging to *Gammaproteobacteria*, NOR5/OM60 group; see the results mentioned above) were located in both the macronucleus and the micronucleus (**Figures 7P–R**).

## Discussion

### Conceptual and Methodological Considerations

To our knowledge, this study is the first to identify the DRB in ciliates that are isolated directly from microbial communities of lab microcosms or the field. We define DRB as an ecologically functional group of bacteria that are able to evade digestion by bacterivorous protists. To characterize DRB, it may be possible to collect these bacteria ingested and subsequently expelled from the protistan cell, since it has been shown that *Salmonella enterica* could be packed in vesicles and released by a soil *Tetrahymena* species (e.g., Brandl et al., 2005). This approach is technically difficult in practice, because such fecal vesicles have not often been observed for other ciliates and there is a high risk of contaminations by environmental bacteria outside the grazer. Alternatively, the bacteria remain inside the predator after starving can be inspected. In this approach, it may be difficult to distinguish these “recently” ingested and these “previously” ingested (i.e., endosymbionts), which both present as the intracellular bacteria. We thus considered both of these groups as DRB because of their similar features in physiology (e.g., indigestibility) and ecological and evolutionary consequences (e.g., adaptation to intracellular life style).

The timespan of starvation may affect our assessment on the richness and composition of DRB assemblages. First et al. (2012) showed that the pathogenic bacterium *Campylobacter jejuni* remained viable after ingestion for 5 h by a freshwater ciliate *Colpoda* sp. During the grazing experiments, *Tetrahymena* cells began expelling vesicles within 1 h after initiation of feeding (Brandl et al., 2005). Starvation for a longer period of time might lead to fewer bacterial species detected in the ciliates (Gong, unpublished data), and a single bacterial species was detected in a paramecium ciliate subjected to starvation for several days (Gong et al., 2014).

The remaining DNA of already digested bacteria might lead to contamination, as DNA-based approaches were applied for detecting DRB in this study. Nevertheless, the DNA of engulfed bacteria that are already digested by host cells of ciliates seems not to be a problem, because these DNA are likely completely hydrolyzed by enzymes in lysosomes during a reasonable period of time. Results on several studies on DNA degradation by lysosomal enzymes are analogous to, and clearly supportive for, this putative intracellular process in protists. Arsenis et al. (1970) found that the calf thymus DNA can be completely degraded into acid-soluble bases by lysosomal extracts of rat liver and Ehrlich ascites tumor cells within 10 h. They also suggested that the hydrolytic action of lysosomes was the highest toward nucleic acids among the various major cellular constituents studied (Arsenis et al., 1970). Another study also found that, when incubated at 37°C, the DNA of apoptotic cells engulfed by macrophages would be degraded by macrophage lysosomal enzymes in a time-dependent manner, and no longer detectable on the gel in a 6-h incubation (Odaka and Mizuochi, 1999). On these bases, we believe the treatment time (12–24 h) was long enough to degrade the DNA of digested bacteria in ciliates, resulting in a minimum probability of this type of contamination. Moreover, this treatment time is not too long to kill the eukaryotic hosts and thus practical in this research.

### High Richness and Assemblage Variations (Beta Diversity) of Digestion-Resistant Bacteria in Ciliates

We detected at least one DRB OTU in each strain and on average three OTUs per strain of the 12 ciliate species. The 40 DRB OTUs affiliated with eight phyla and about 14 families were found in this study, suggesting that there is a collectively high phylogenetic diversity of DRB in these bacterivorous protists. Nevertheless, since we used approaches of clone libraries and Sanger sequencing of 16S rRNA genes, which are known to have limited sampling depths to recover rare phylotypes in complicated microbial communities, the OTU-level diversity of DRB in this study is more likely attributed to these most abundant or commonly occurred. Future studies with high-throughput sequencing may reveal higher phylogenetic diversities with more rare phylotypes of various phyla in the DRB assemblages.

Relative to the well-known high diversity and complicated structure of bacterial communities in the natural aquatic environment, the DRB assemblages in ciliates were apparently much simplified. This partly reflects the selective feeding behavior of ciliate grazers, through which some bacterial populations in the community are preferably engulfed. Selective ingestion of bacteria by ciliates depends on bacterial size (Gonzalez et al., 1990; Posch et al., 2001), growing phase (Sherr et al., 1992), ratios of C:P and N:P (Gruber et al., 2009), and motility (Sherr and Sherr, 2002). It is highly likely that only a fraction of ingested bacterial preys could have the ability to survive ciliate digestion, though currently we know little about which groups of ingested bacteria can be completely digested.

Only a few DRB OTUs were shared among the 12 ciliate species and no OTUs were common in these two strains of *Condylostoma spathiosum*, showing considerable assemblage variations (beta diversity) among ciliate species and even between populations, suggesting there is a high “species”-level diversity of DRB to be discovered. The null hypotheses that there were effects of habitat and isolation source of hosts on the DRB composition at the family level were all rejected. Despite this, we insist that it is premature to rule out the role of environment in structuring the DRB in these protists, as other ecological and physiological factors may not considered in the present study. Boenigk et al. (2001) found that preculture conditions influenced the ingestion and digestion process of HNFs. There are species-specific differences in, and an effect of physiological state on, ciliates’ gazing and digestive ability upon bacteria (Christaki et al., 1998; Weisse, 2002). Also rejected was the hypothesis on the effect of class-level taxonomy of ciliates. However, it should be noted that the two species of *Pseudokeronopsis* were placed closely to each other in the clustering plot, so were the two *Condylostoma* strains (**Figure 6**). This suggests the selection of DRB may be relatively stable at a lower taxonomic level (e.g., genus or species) of the hosts. Our last hypothesis, the DRB composition was different between free-living and endosymbiotic ciliates, was statistically supported. All these imply that the changes of DRB assemblages may be linked with both ecological traits of the host and the environment, but the key factors and their relative importance remain to be revealed.

### Novel Diversity and Rare Taxa of Bacteria Associated with Ciliates

Among 40 bacterial OTUs we detected, about 43% (17 OTUs) had a closet match of 16S rRNA gene sequences of identified species available from the GenBank with a similarity < or = 97%, even for some culturable taxa such as *Alteromonadaceae, Pseudoalteromonadaceae, Vibrionaceae*, and *Enterobacteriaceae*. These indicate that there are a great number of yet-to-be-described bacteria associating with protists. Furthermore, our previous study demonstrated the association between OD1 bacteria and a freshwater ciliate (Gong et al., 2014). This uncultivated rare bacteria-protist association was observed again in the present study (e.g., OD1 and GN02), highlighting that many of these rare bacteria (also known as “microbial dark matter”) are of digestion-resistant and symbiotic nature in the environment (Brown et al., 2015; Nelson and Stegen, 2015).

### Predominance of *Gammaproteobacteria* and *Alphaproteobacteria* in the Digestion-Resistant Bacteria Assemblages

Despite of considerable species-level variations, a structuring pattern of DRB at higher taxonomic ranks was evident: in terms of either the number of OTU or the relative abundance, members of *Gammaproteobacteria* and *Alphaproteobacteria* were the most abundant in the DRB assemblages. This result may more or less bias to ciliates of marine origin, since we studied more marine than freshwater ciliate species. However, a gammaproteobacterial OTU related to *Aestuariibacter halophilus* (*Alteromonadaceae*) comprised of 96% sequences in the clone library of the DRB of the freshwater *P. aurelia*, which is still supportive of the pattern mentioned above. Our result is in coincidence with two studies demonstrating a predominance of gamma- and alphaproteobacteria in bacterial 16S rRNA gene sequences recovered from a number of heterotrophic and mixotrophic marine protists (mostly flagellates) (Martinez-Garcia et al., 2012), and in the marine ciliate *Euplotes focardii* (Pucciarelli et al., 2015). Although their experiments were not designed for studying DRB in these protists, the procedures of sample preparation and cell sorting by flow cytometry must have taken some time, which allowed these protistan cells to digest bacteria in their food vacuoles, so that the digestion-resistant populations were more likely retained and detected. As such, the resistance of gamma- and alphaproteobacteria to digestion seems to be applicable for a broad range of marine protistan grazers, which may reflect some biological properties of these two groups of marine origin.

The resistance of marine gamma- and alphaproteobacterial to protistan digestion may be related to their protein secretion systems. The secretion systems in Gram-negative bacteria are often important virulence factors, comprising a diversity of proteinaceous machines to translocate secreted proteins from cell cytosol to the extracellular space or across the host cell membrane. Among the known six general types of these systems in Gram-negative bacteria, only the types III, IV and VI secretion systems (T3SS, T4SS, and T6SS) can deliver proteins further across the plasma membrane of the host in a contact-dependent manner (Tseng et al., 2009). The T4SS are capable of transporting DNA in addition to proteins into many eukaryotic cells and bacteria, suppressing host’s defense mechanisms and facilitating intracellular colonization (Christie and Cascales, 2005). Some bacteria (e.g., *Legionella pneumophila*) require the T4SS to become resistant to lysosomal degradation (Shintani and Klionsky, 2004). Using the amoeba *Dictyostelium discoideum* as a model host system, the T6SS was first identified in *Vibrio cholera* (Pukatzki et al., 2006). The genes encoding T6SS components are widely present in about 25% sequenced genomes of bacteria, mostly of proteobacterial pothogens (e.g., *Pseudomonas aeruginosa, Francisella tularensis, and Burkholderia mallei*), with the gammaproteobacteria being the most widely representated (Shrivastava and Mande, 2008). According to a metagenomic survey of virulent genes in marine bacteria, among all detected secretion systems, the T6SS and T4SS were the most abundant, and were mostly found in gamma- and alphaproteobacteria, respectively (Persson et al., 2009). Recent studies suggested that T6SS may play an important role in promoting a symbiotic relationship between some bacteria and mammals (Chow and Mazmanian, 2010), and in mediating communication between bacteria and eukaryotic hosts (Jani and Cotter, 2010). Besides T6SS or T4SS, the genomes of several marine *Gamma-* and *Alphaproteobacteria* strains possess type III secretion systems (T3SS) (Persson et al., 2009), which has been demonstrated to promote survival of *V. parahaemolyticus* in the interaction with diverse protists (Matz et al., 2011).

### *Alteromonadaceae, Pseudoalteromonadaceae*, and *Vibrionaceae* as the Most Frequently Occurring Digestion-Resistant Bacteria in Ciliated Protists

In this study, rice grains were added to enrich bacterial prey to maintain seven ciliated species (**Table 1**), in which DRB phylotypes affiliated with *Alteromonadaceae, Pseudoalteromonadaceae*, and *Vibrionaceae* appeared much more abundant than those in the ciliates obtained directly from field samples. This suggests that the abundance of these digestion-resistant gammaproteobacterial lineages is probably related to the addition of a carbon source. In fact, previous studies demonstrated that the enrichment of dissolved organic matter selected for these *Gammaproteobacteria* lineages (Allers et al., 2007; Alonso-Saez et al., 2009; McCarren et al., 2010; Kelly et al., 2014). While *Gammaproteobacteria* lineages might resist grazing by nanoflagellates in the microcosms enriched with glucose by forming filamentation and aggregation (Alonso-Saez et al., 2009), the large particle size seems not a problem for most ciliate species investigated in this study. For example, the cultured hypotrich ciliates (class Spirotrichea, e.g., *Diophrys scutum, Hemigastrostyla elongata, Pseudokeronopsis carnea*, and *Ps. flava*) have large cell sizes (150–250 μm in length) and wide oral openings (40–60 μm), which facilitate engulfment of large-sized particles including bacterial filaments, aggregates and dinoflagellate, heterotrophic flagellate, and diatom species. Moreover, when the nanoflagellate grazers are eaten by the ciliates, indigested bacteria in nanoflagellates may retain in ciliate cells. Therefore, the abundance of *Alteromonadaceae, Pseudoalteromonadaceae*, and *Vibrionaceae* as DRB in ciliates is likely related to the eutrophic condition during cultivation of ciliates, and may reflect the DRB in the microbial food webs of the studied systems.

Our finding on the indigestibility of *Alteromonadaceae, Pseudoalteromonadaceae*, and *Vibrionaceae* is relevant to, and may be accountable for, some previously observed ecological phenomena. Members of these groups are well known for pursuing a “feast-or-famine” growth strategy in marine bacterioplankton, that is, they are able to maintain high ribosome levels during starvation and become rapidly enriched when organic matter are amended, but usually rare *in situ* (Eilers et al., 2000). In marine confinement experiments, Schäfer et al. (2000) found that *Alteromonas*-like phylotypes dominated the bacterial assemblages during and after the peak of protistan grazing pressure; they supposed that these populations perhaps have an inedible morphotype to survive protistan grazing. Their suggestion is apparently supported by our finding that these gammaproteobacteria can be ingested but may not be digested by protistan grazers. However, Beardsley et al. (2003) observed that an overproportional decline of *Alteromonas, Pseudoalteromonas*, and *Vibrio* during the phase of HNF regrowth. A possible explanation is that the indigestibility of these bacteria might be species- or strain-dependent, as ecotypes of *Alteromonas* or *Pseudoalteromonas* could have substantially different gene content and metabolic potentials (Ivars-Martinez et al., 2008; Qin et al., 2011), and it has been shown that the T6SS can be horizontally transferred between some marine vibrios (Salomon et al., 2015). Alternatively, if these phylotypes were indeed indigestible, then their massive mortality after blooming could be due to the viral lysis, which is another important top–down control on bacterial mortality. Both these two hypotheses are needed to be further tested in order to better understand the causes and the consequences of bacterial indigestibility in a microbial loop context.

### Activity of Digestion-Resistant Bacteria

The FISH assays targeting bacterial rRNA genes illustrated that at least some of these DRB were still active after starvation. Nevertheless, it should be cautious that there was also diffuse fluorescence of Cy3-probe signals inside some ciliate specimens, indicating non-bacterial-cell bindings of targeted rRNA molecules. This is probably due to the remaining of rRNA of digested bacterial cells. According to Arsenis et al. (1970) who comparatively studied the degradation of DNA and RNA by lysosomal extracts, the complete degradation of RNA was considerably slower (within 60 h) than that of DNA (within 10 h). Considering these analogous situations, we assume that, although morphology of bacterial cells were not recognizable in some intracellular regions, their RNA (including ribosomal RNA) might not be completely degraded during the treatment of ciliate cells for 12 to 24 h, thus gave rise to the diffuse fluorescence after FISH. This may cause some biases on inferring activity of DRB in ciliates. Both diffuse rRNA-targeted Cy3 signals and rDNA-based identity of *Alteromonadaceae* were obtained from *Paramecium aurelia* (**Figures 7G–I**), suggesting the indigestibility of some DRB species might be strain-or phylotypes-dependent. This recalls intra-species variations of genomic contents and inter-species horizontal transfer of genes encoding protein secretion systems (Ivars-Martinez et al., 2008; Qin et al., 2011; Salomon et al., 2015). Further studies using more dedicated tools are needed to reveal their activity and function inside protistan cells and to assess the stain-level capability of digestion resistance.

## Conclusion

Effective protistan grazing on bacteria relies on the success of two successive steps, ingestion and digestion, of which the latter has been rarely studied for nanoflagellates and ciliates from an ecological perspective. The causes and consequences of indigestibility of bacteria in the microbial loop remain elusive. For the first time, we explicitly characterized the ingested but not digested bacteria in a range of ciliate species. Our study reveals a snapshot of DRB diversity and structure, in which the number of investigated protists is apparently lower in comparison with the known enormous protistan diversity in nature. Despite this, we found a collectively high phylotype richness and large composition variations of DRB among protistan species, supporting the previous hypothesis that resistance to digestion is a widespread mechanism in natural bacteria (Jürgens and Güde, 1994). Previously, several structural and food quality traits of bacteria have been suggested for bacterial indigestibility. We are the first to find indications of a taxonomical strait of DRB of marine origin and suggest that it may be relevant to the putative symbiosis-promoting secretion systems that exist widely in the genomes of marine bacteria.

The findings of the high indigestibility of several opportunistic and copiotrophic groups not only provide a clue to explain that the mortality of these rapid growing bacteria in marine bacterioplankton, but also raise many further ecological questions. For example, given that there is a high diversity of bacteria capable of escaping protistan predation, why many of these bacterial winners are usually not abundant in the marine bacterioplankton? Does viral lysis play a significant role in killing these DRB? Did we overestimate protistan bacteriovory (feeding/grazing rates) by some traditional approaches, in which short-time uptakes of fluorescent labeled beads or model bacterial strains were estimated, assuming all the ingested fluorescent prey surrogates will be digested? How do the presence and expression of bacterial secretion systems impact the microbial ecology and function? It seems that indigestibility or limited digestibility of bacterial species is among the key factors regulating the interactions among bacteria, protists, and viruses. In the near future, multi-disciplinary approaches may be used to unveil the molecular mechanisms and test the ecological hypotheses concerning DRB in diverse and changing ecosystems.

## Author Contributions

JG and YQ conceived and designed the experiments; YQ and SZ performed the experiments; JG, YQ, SZ, RF, LS, XZ, and QZ analyzed data; JG, YQ, SZ, XZ, and QZ contributed to reagents/materials; JG and YQ wrote the paper.

## Conflict of Interest Statement

The authors declare that the research was conducted in the absence of any commercial or financial relationships that could be construed as a potential conflict of interest.

## Footnotes

1 http://itol.embl.de/
